# Specific determination of hepatitis B e antigen by antibodies targeting precore unique epitope facilitates clinical diagnosis and drug evaluation against hepatitis B virus infection

**DOI:** 10.1080/22221751.2020.1862631

**Published:** 2021-01-17

**Authors:** Shao-Juan Wang, Zi-Min Chen, Min Wei, Jia-Qi Liu, Zong-Lin Li, Tian-Shu Shi, Sheng Nian, Rao Fu, Yang-Tao Wu, Ya-Li Zhang, Ying-Bin Wang, Tian-Ying Zhang, Jun Zhang, Jun-Hui Xiong, Shu-Ping Tong, Sheng-Xiang Ge, Quan Yuan, Ning-Shao Xia

**Affiliations:** a State Key Laboratory of Molecular Vaccinology and Molecular Diagnostics, School of Public Health, Xiamen University, Xiamen, People’s Republic of China; b National Institute of Diagnostics and Vaccine Development in Infectious Diseases, School of Life Science, Xiamen University, Xiamen, People’s Republic of China; c Xiamen Innodx Biotech Co., Ltd., Xiamen, People’s Republic of China; d Liver Research Center, Rhode Island Hospital and Warren Alpert Medical School of Brown University, Providence, RI, USA

**Keywords:** Hepatitis B virus, Hepatitis B e antigen, monoclonal antibody, cccDNA surrogate, immunoassay

## Abstract

Hepatitis B e antigen (HBeAg) is a widely used marker both for chronic hepatitis B (CHB) clinical management and HBV-related basic research. However, due to its high amino acid sequence homology to hepatitis B core antigen (HBcAg), most of available anti-HBe antibodies are cross-reactive with HBcAg resulting in high interference against accurate measurement of the status and level of HBeAg. In the study, we generated several monoclonal antibodies (mAbs) targeting various epitopes on HBeAg and HBcAg. Among these mAbs, a novel mAb 16D9, which recognizes the SKLCLG (aa −10 to −5) motif on the N-terminal residues of HBeAg that is absent on HBcAg, exhibited excellent detection sensitivity and specificity in pairing with another 14A7 mAb targeting the HBeAg C-terminus (STLPETTVVRRRGR, aa141 to 154). Based on these two mAbs, we developed a novel chemiluminescent HBeAg immunoassay (NTR-HBeAg) which could detect HBeAg derived from various HBV genotypes. In contrast to widely used commercial assays, the NTR-HBeAg completely eliminated the cross-reactivity with secreted HBcAg from precore mutant (G1896A) virus in either cell culture or patient sera. The improved specificity of the NTR-HBeAg assay enables its applicability in cccDNA-targeting drug screening in cell culture systems and also provides an accurate tool for clinical HBeAg detection.

## Introduction

Chronic hepatitis B virus (HBV) infection is still a major cause of end-stage liver diseases worldwide including liver cirrhosis (LC) and hepatocellular carcinoma (HCC). According to recent studies, there are more than 290 million people persistently infected with HBV, which results in nearly 1 million deaths annually primarily due to HBV-induced LC and HCC [1, 2]. Chronic HBV infection usually presents a complex and long-term natural history which is characterized by a series of serum and liver markers, including hepatitis B e antigen (HBeAg) status, hepatic inflammation markers (liver enzyme, histologic necroinflammatory scoring) and serum HBV DNA levels [3]. Among these markers, HBeAg is non-particulate secretory protein and produced very early in the life cycle of HBV. The HBeAg and hepatitis B core antigen (HBcAg) are two alternative in-frame translation products of HBV precore/core open reading frame (PreC/C ORF). HBcAg is a translation product of the core gene alone (183aa for most HBV genotypes) using pregenomic RNA (pgRNA) as its mRNA, which lacks precore AUG at its 5' end. HBeAg is derived from translation product of the entire precore/core ORF, and the corresponding precore mRNA (pcRNA; slightly longer than pgRNA) has the precore AUG covered at its 5' end. Of the extra 29aa encoded by the precore region, the first 19aa serve as the signal peptide targeting the precore/core protein into the secretory pathway [4, 5]. Cleavage of the signal peptide followed by the arginine-rich C-terminus between residues 149 and 154 by proprotein convertase furin generates mature HBeAg. Therefore, HBeAg has 10 extra residues at its N-terminus (NTR, aa −10 to −1, SKLCLGWLWG) than HBcAg but lacks C-terminal 29–34 residues [6, 7]. The 183-aa HBcAg comprises a capsid-forming region called assembly domain (AD, aa 1–149) and a basic arginine-rich C-terminal domain (CTD, aa 150–183) for viral DNA and RNA binding [8]. The HBcAg forms the building block of viral capsid which encloses HBV DNA and polymerase, and it also mediates the interaction with inner domain of viral surface proteins therefore contributing to viral envelopment.

Although HBeAg is a nonstructural protein, several studies suggested it plays multitude roles in the establishment and progression of chronic HBV infection by modulating the host immune response [9]. Secreted HBeAg preferentially elicits non-inflammatory Th2 cells and deletes inflammatory Th1 cells by Fas-associated apoptosis [10]. In addition, studies with mouse models have shown that HBeAg plays a role in the impairment of CD8^+^ cytotoxic T lymphocytes following vertical transmission of HBV [11]. The HBeAg also suppresses the Toll-like receptor (TLR) signalling pathway by interacting with the Toll/interleukin-1 receptor (TIR)-containing proteins Mal and TRAM and disrupting homotypic TIR to TIR interactions critical for TLR signalling [12]. More recently, HBeAg was also shown to suppress the IFN/JAK/STAT signalling pathway by stimulating the expression of suppressor of cytokine signalling 2 (SOCS2) [13].

As its immune modulating effects, serological HBeAg status is regarded as an important parameter in HBV clinical diagnosis and the immunoassays detecting HBeAg were routinely used in clinical settings for several decades. Serum HBeAg-positive chronic hepatitis B (CHB) patients usually have significantly higher viremia levels, also higher HCC risk than HBeAg-negative patients. HBeAg seroconversion (the loss of serum HBeAg accompanied by the development of anti-HBe antibodies) is considered as an important milestone of favourable outcome and good progression of treatment response. Patients who undergo HBeAg seroconversion are more likely to experience improved long-term outcomes, including disease remission, a lower incidence of cirrhosis and HCC, increased rates of survival. Moreover, the recommendation strategies for treatment and follow-up are quite different in most of CHB Clinical Practice Guidelines between HBeAg-positive and -negative patients [3].

Despite the importance of HBeAg as an indicator for CHB clinical management, current HBeAg immunoassays are not ideal because the existing ones cross-react with HBcAg, the HBeAg homologue [14]. Because HBeAg shares about 152 aa sequence with HBcAg, most of antibodies used in current HBeAg assays are not absolutely unreactive with HBcAg [8]. It is generally assumed that the naked HBcAg capsid does not release into bloodstream unless HBV-infected hepatocytes are damaged. Therefor little attention is paid to the concern of the Influence of naked HBcAg capsid on HBeAg assay. However, several recent studies revealed HBV-infected hepatocytes secrete not only enveloped virions but also naked HBcAg capsids in a non-lytic manner [15]. Although it was suggested that naked HBcAg capsids in blood circulation were bound with Anti-HBc antibodies, we hypothesized that some cross-reactive epitopes on HBcAg were still accessible for capture antibody that is used in current HBeAg immunoassays. Theoretically, an ideal HBeAg assay should be based on antibodies specific to NTR region unique to HBeAg and therefore absolutely avoid the potential interference of HBcAg. However, no such HBeAg assay has been reported so far. In this study, we generated high-affinity monoclonal antibodies (mAbs) which recognize epitopes in HBeAg unique NTR region and developed a highly specific chemiluminescent microparticle HBeAg immunoassay based on these new mAbs.

## Materials and methods

### Generation and characterization of mAbs specific for HBeAg and HBcAg

**Figure 1.**
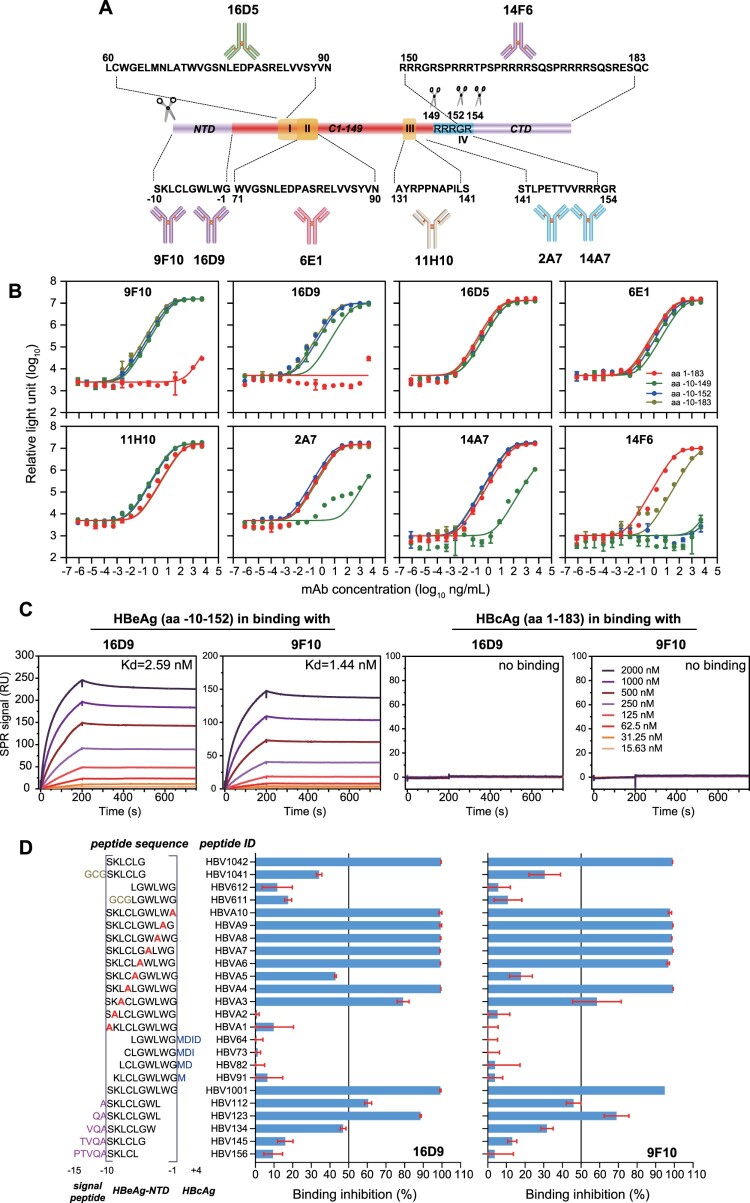
**Generation and characterization of mAbs specific for precore/core proteins**. (A) Schematic diagram of mAbs which generated in this study and their binding epitopes on precore/core proteins. (B) Binding activities of mAbs to recombinant core/precore proteins of aa (−10)-149, aa (−10)-152, aa (−10)-183, and aa1–183 by indirect chemiluminescent immunoassay. (C) SPR sensorgrams showing the binding kinetics for the proteins of aa (−10)-152 and aa1–183 to immobilized NTR-mAbs of 9F10 and 16D9. Coloured lines represented a global fit of the data at known concentrations using a 1:1 binding model. (D) Peptide competitive ELISA-binding assay to analysis the accurate targeting epitopes of the two NTR-mAbs. The sequences of peptide competitors were showed on the left *Y*-axis. Peptide competitors with an over 50% inhibition indicated the corresponding peptides contain the binding motif of the NTR-mAbs.

Recombinant proteins containing aa (−10)-149, aa (−10)-152, aa (−10)-183, and aa 1–183 of HBV precore/core ORF were expressed in *E. coli* BL21 cells following previously described [16]. These proteins were purified by ion-exchange chromatography. The purified proteins were used as immunogens to stimulate mice (BALB/c) to produce mAbs using hybridoma technology. A total of 8 mAbs, including 16D9, 9F10, 2A7, 14A7, 6E1, 16D5, 11H10 and 14F6, were used in this study (Figure 1A). Among them, the mAbs of 16D5 and 11H10 have been described in our previously studies, and the remaining ones were first reported in this study. To determine the binding epitopes of these mAbs, several peptides derived from HBcAg/HBeAg were synthesized (Sangon Biotech) to test the mAb reactivity. Affinity of the mAbs was determined by Surface Plasmon Resonance (SPR) on Biacore T200 using Mouse Antibody Capture Kit and S Sensor Chip CM5 (GE Health).

### Cell cultures and HBV plasmid transfections

HepAD38 cell line, that is stably transfected with a HBV 1.1-fold genome of genotype D under tetracycline (Tet) response promoter, was cultured as previously described [17, 18]. Briefly, the cells were grown in the presence of 1 μg/mL tetracycline and 400 μg/mL G418 in Dulbecco's modified eagle's medium (DMEM) supplemented with 10% fetal bovine serum (FBS). To initiate the productions of pgRNA, replicative DNA, HBcAg and HBeAg, tetracycline was removed from culture medium when cells reached about 80% confluence. Supernatants were collected for evaluations of mAbs and immunoassays. To produce HBeAg of various genotypes, a serial of HBV 1.3-fold or CMV-driven 1.1-fold genome plasmids with genotypes A-I (detailed information was shown in supplemental Table 1) or with precore G1896A mutation were transfected into HepG2 cells or Huh7 cells using X-tremeGENE HP DNA Transfection Reagent (Roche).

### Antibody conjugations and novel HBeAg immunoassay

The HBeAg-NTR specific mAb of 16D9 was covalently coupled to carboxylate-modified magnetic beads (JSR company, Tokyo, Japan) through EDC-NHS esterification. Preparation of the acridinium ester (AE)-labelled mAbs was performed following previously reported method [19]. For HBeAg assay, 50 μL of sample was first mixed with 10 μL reducing buffer (containing 0.5 M of 1-thioglycerol and 8.0 M of urea in 50 mM Tris–HCl buffer, pH 8.0) and 50 μL 16D9-immobilized magnetic beads, followed by an incubation at 37°C for 15 min. After a washing step, the beads were further incubated with 50 μL of AE-labelled mAbs at 37°C for 10 min. Then, the beads were washed again and treated with 100 μL of pre-trigger solution. Finally, after removal of pre-trigger solution, 100 μL of trigger solution was loaded into each tube and the relative light unit (RLU) was measured immediately by using photomultiplier detector. The whole detection procedure was automatically completed on a Caris 200 fully automated chemiluminescence analyser (Innodx, Xiamen, China).

### Immunoprecipitation-western blot, immunofluorescence and southern blot

To analyse the form of precore/core proteins in HBV cell cultures and serum samples of CHB patients, an immunoprecipitation-based western blot (IP-WB) assay was used. In this assay, 500–1000 μL cell culture medium or 200 μL patient's serum were first mixed with 50 μg antibody-immobilized magnetic beads and incubated at room temperature for 2–4 h. The beads were separated and washed by using magnetic rack, and then incubated with 40 μL lysis solution containing 2% SDS and 1% β-2 me (2-hydroxy-1-ethanethiol) at 98°C for 20 min to release immunoprecipitated antibody-bound proteins. Subsequently, the beads were removed and the whole supernatant was subjected to SDS-polyacrylamide gel electrophoresis (SurePAGE, Genscript, China). Then, the proteins were transferred onto a nitrocellulose membrane and stained with HRP-conjugated antibodies. After the unbound antibody-HRP conjugates were washed away, the membrane was incubated with a SuperSignal West Femto Substrate (Thermo Scientific) and detected by using Fusion FX7 Spectra imaging platform (Vilber, France). HepG2-N10 (a HepG2-derived cell line that stably integrated with a HBV 1.3-fold genome of genotype Ae) and HepG2 cells were used to test detection performance of the new mAbs using immunofluorescence according previously described experimental protocol [20].

### Drug treatment on HepAD38 and cccDNA quantifications

HepAD38 cells were used to test the performance of new HBeAg assay in drug screening. Entecavir (ETV), Morphothiadin (Mor) and RG7834 were purchased from MedChemExpress. For cccDNA detections, extrachromosomal DNA of cells was extracted following modified Hirt procedure [21]. The quantification of cccDNA was performed using qPCR method as previously described [22]. The 5'-TGCACTTCGCTTCACCT (forward) and 5'-AGGGGCATTTGGTGGTC (reverse) were used as cccDNA specific primers, and the 5'-FAM-ACCGTGAACGCCCACCGAATGTTGC-BHQ1 was used as probe. Mitochondria DNA (mtDNA) was detected for internal reference using the primers of 5'-CCCAGCTACGCAAAAT (forward), 5'-AATGCGGTAGTAGTTAGGATA (reverse) and the probe of 5'-HEX-CATACTCCTCAATTACCCACATAG-BHQ. The qPCR was performed using the Premix Ex Taq on LightCycler® 96 Real-Time PCR System as the following reaction procedure: 95°C for 5 min then 45 cycles of 95°C for 30 s and 55°C for 40 s.

### Clinical samples

Plasma samples from different individuals infected with various HBV genotypes were purchased from SeraCare (BBI Diagnostics, supplemental Table 2). Besides, a total of 181 serum samples from an untreated cohort of Chinese patients with chronic HBV infection (genotype B or C) were also involved in this study to compare the performance of different HBeAg assays. These patients were positive for HBsAg at last for 6 mouths, and those with co-infection of HIV/HCV or other co-existent autoimmune or metabolic liver disease were excluded. The cohort included 49 disease-free HBV carriers, 75 CHB patients, 24 LC patients and 33 HCC patients. The clinical and virological characteristics of these patients were listed in supplemental Table 3. Viral genome were obtained PCR using LA Taq polymerase (Takara) and PCR products were directly sequenced on ABI Prism 3130X automatic genetic analyser following methods described elsewhere [23]. Besides the cohort samples, a total of 464 serum samples from HBV-free healthy donors that collected from Xiamen Blood Centre were also involved to evaluate the specificity of the new assay. The study was approved by the institutional review board of School of Public Health in Xiamen University in accordance with the Declaration of Helsinki. Written informed consent was obtained from all patients.

### Laboratory measurements of blood samples

Serum HBsAg levels were determined by Abbott Architect HBsAg assay with a dynamic range of 0.05–250 IU/mL. Samples with HBsAg levels of >250 IU/mL were retested at dilutions of 1:500 or 1:1000. Serum HBeAg were measured by Abbott Architect assay, Roche Elecsys assay and the new assay based on NTR-specific mAb (termed NTR-HBeAg assay). Anti-HBe was measured by using Abbott Architect Anti-HBe assay. Quantitative anti-HBc levels were determined using a double-sandwich assay as previously described [16]. For serum HBV genome sequencing, complete HBV genomes were amplified by a high-fidelity polymerase and the PCR products were directly sequenced using the previously reported method [23].

### Statistical analysis

The unpaired *t*-test or Kruskal–Wallis test was used to compare continuous variables, and the Mantel-Haenszel *χ*^2^ test or Fisher's exact test was used for categorical variables. Linear regression models and Pearson correlation coefficient (*r*) were used to determine strength and direction between two quantitative variables. An analysis with a two-side *p* < 0.05 was considered statistically significant. SPSS software V22.0 was used for the statistical calculations.

## Results

### Characterizations of mAbs that recognizes different epitopes of HBeAg and HBcAg

The differences between capsid-building HBcAg and secreted HBeAg are that the latter has a longer N-terminus and shorter C-terminal tail. Previous studies suggested the signal peptide of HBeAg precursor is removed by signal peptidase at cleavage site between (−11) Ala and (−10) Ser and the removal of its CTD is predominantly processed by furin-like proprotein convertases between 151Arg and 154Arg. Therefore, we generated two mAbs (16D9 and 9F10) against the ten N-terminal residues (NTR, aa (−10)-(−1), SKLCLGWLWG) for specifically recognizing HBeAg (Figure 1A). Besides the NTR-mAbs, we also developed two mAbs (2A7 and 14A7) that bind epitopes surrounding CTD furin cleavage site (FCR, aa141-152). To specifically differentiate HBcAg, 14F6 mAb which is specific to CTD region (recognizes PRRR motif) was developed. Additionally, three mAbs of 6E1 (targeting aa71–90), 16D5 (targeting aa60–90) and 11H10 (targeting aa131–141) that recognize HBeAg and HBcAg sharing sequence were also involved as analytical tools.

We first tested the binding activities of the mAbs mentioned above to the recombinant proteins containing aa (−10)−149, aa (−10)−152, aa (−10)−183 and aa1–183 of HBV precore/core proteins by indirect immunoassay. As expected, the mAbs of 16D5, 6E1 and 11H10, which recognize the HBcAg and HBeAg sharing region did not show significant difference in binding to these proteins (Figure 1B). The FCR-mAbs, 2A7 and 14A7, showing specific reactivity to synthetic peptide of aa141–154, presented strong binding activities to the proteins of aa (−10)−152, aa1–183 and aa (−10)−183 and dramatically decreased binding to the protein of aa (−10)−149. Therefore, the 2A7 and 14A7 mAbs recognized an epitope surrounding aa149–152. The NTR-mAbs of 9F10 and 16D9 exhibited strong binding activities to the proteins of aa (−10)−149, aa (−10)−152 and aa (−10)−183, but not to the aa1–183 protein. Further SPR analyses demonstrated the binding affinities to the aa (−10)-152 protein was 1.44 nM for 9F10 and 2.59 nM for 16D9, whereas no binding was detected to the aa1–183 protein (Figure 1C). We further investigated the accurate targeting epitopes of the two NTR-mAbs using competitive ELISA-binding assay with a series of synthetic peptides. The results revealed that both mAbs bind to an identical SKLCLG motif, which is located on aa(−10)–aa (−5) region of precore/core proteins (Figure 1D). Mutational analyses suggested the (−10) Ser and (−9) Lys are crucial for the binding of the two mAbs.

### Evaluation of various mAbs in detecting HBeAg and HBcAg

**Figure 2.**
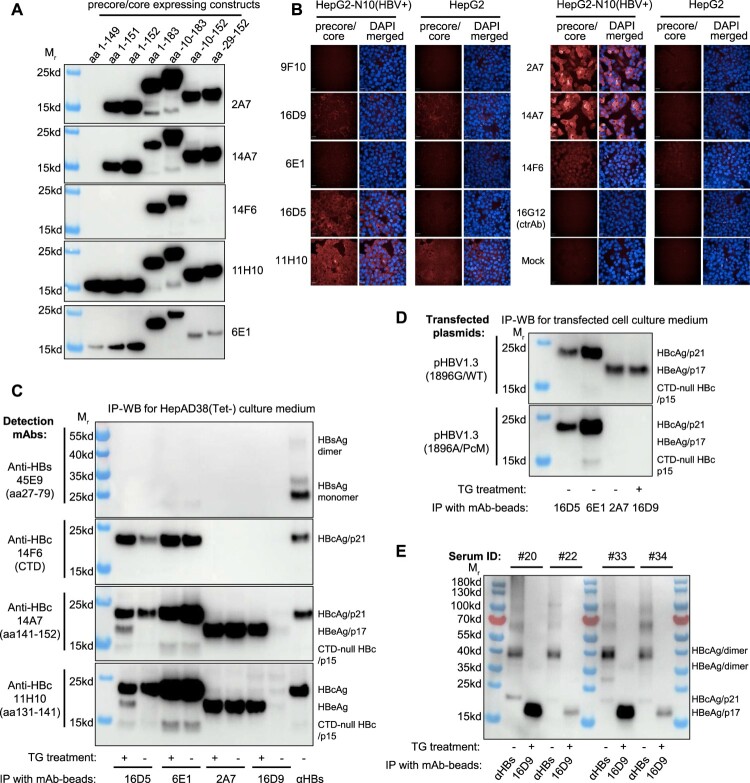
**Performance of the mAbs in detecting precore/core proteins.** (A) Western blots using different mAbs to detect precore/core derivatives in cell lysates that transfected with various expressing constructs. (B) Immunofluorescence using different mAbs to detect intracellular precore/core proteins in HBV-integrated HepG2-N10 cells. (C) IP-WB analyses using supernatants of HepAD38 cells to evaluate the capture-binding potentials of different mAbs. The precore/core proteins in cell culture supernatants were captured by magnetic beads immobilized with different mAbs. The immunoprecipitation samples were analysed by immunoblotting using HPR-linked mAbs of 45E9 (an anti-HBs mAb), 14F6, 14A7 and 11H10. Supernatants were used in untreated condition or pretreated with TG. (D) IP-WB analyses of the HBeAg presented in patients’ sera using 16D9-conjuated beads. HRP-linked 14A7 was used to detection. For panels (C) and (D), beads that conjugated with polyclonal anti-HBs antibody (αHBs) were used as a control to capture the intact HBV virions. (E) Western analyses of capture-binding potential of various mAbs to precore/core derivatives in sera from HBeAg-positive CHB patients.

We evaluated the performance of these mAbs in western blot detecting precore/core derivatives from Huh7 cells transfected with various constructs. Among them, the mAbs of 16D9, 9F10 and 16D5 could not react with SDS-denatured targets (data not shown), whereas the 2A7, 14A7, 14F6, 11H10 and 6E1 mAbs showed specific bands (Figure 2A), suggesting these mAbs bind to linear epitopes. The tests comparing HBV-N10 (a stable HepG2 cell line stably expressing HBV 1.3-fold genome) and HepG2 cells demonstrated the 16D5, 2A7, 14A7 and 14F6 could be used for immunofluorescence (IF) detection of intracellular HBcAg, whereas the remaining mAbs did not show specific signal in IF detection(Figure 2B). To test the binding potentials of NTR-mAbs and FCR-mAbs to cell culture derived viral proteins, supernatants of HepAD38(Tet-) cells were immunoprecipitated by 16D5, 6E1, 2A7, and 16D9 mAbs-conjugated beads, respectively. To avoid the potential mAb-binding interference due to intramolecular cysteine bridges of HBeAg, the supernatants were tested in the presence or absence of α-tyioglycerrol (TG). As shown in Figure 2(C), only anti-HBs-beads captured surface proteins in associating with HBcAg. For TG-treated cell culture supernatants, three typical bands of precore/core derivatives, including p21 (HBcAg), p17 (HBeAg) and p15 (CTD-cleaved HBcAg), were detected in 16D5-immunoprecipitated samples by 14A7 or 11H10 mAb (Figure 2C). As expected, only the p21 could be detected by the CTD-mAb of 14F6, whereas neither p17 nor p15 band was revealed by 14F6. For non-reduced samples, 16D5 could only capture the HBcAg/p21. The 6E1 mAb, which mainly binds with p21 and p15 instead of p17, showed little difference in between native and reduced samples. Interestingly, the FCR-mAb of 2A7 exhibited p17-specific and TG treatment-independent capture capability, although it reacted well with SDS-denatured various precore/core derivatives (Figure 2C). This result suggested the 2A7/14A7-targeted FCR epitope is not exposed on the surface of core capsid and is inaccessible in native condition, and it is only present in the HBeAg/p17 protein. The NTR-mAb of 16D9 also showed specific capture capability to HBeAg/p17 protein, but its binding seems to be TG dependent. In a comparison of supernatants from HepG2 cells transfected with pHBV1.3 plasmids of precore wild-type and 1896A mutant (a common HBeAg-minus mutant), the HBeAg/p17 blot bands were successfully detected in 2A7 and 16D9-immunoprecipitated samples transfected with wild-type HBV, but not in that transfected with 1896A mutant (Figure 2D). Moreover, the IP-WB tests using serum samples (*n* = 4) from HBeAg-positive CHB patients also found that the 16D9 could specifically and efficiently capture HBeAg/p17 (Figure 2E). Together, these results demonstrated that the NTR-mAb of 16D9 could capture both cell culture-derived and patient serum-derived HBeAg in a highly specific manner.

### Establishment of a novel HBeAg immunoassay based on NTR-mAb

**Figure 3.**
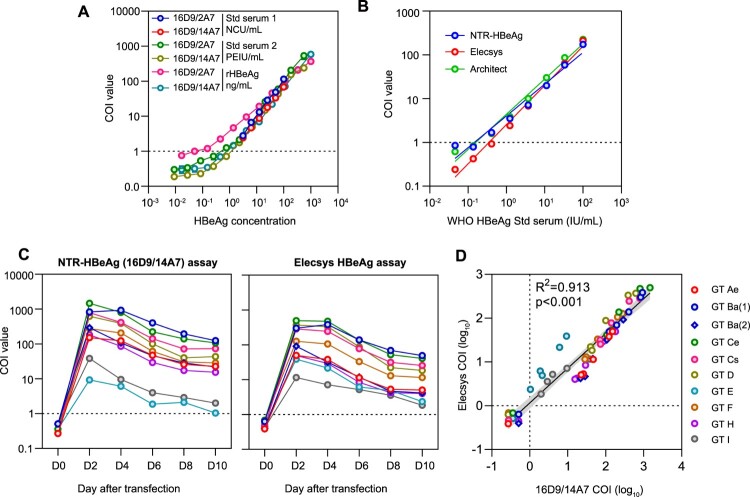
**Evaluation of the analytical performance of the NTR-HBeAg assay.** (A) Evaluation of the analytical sensitivity of the NTR-HBeAg prototype assays (with 16D9 in pairing with 14A7 or 2A7) using serial diluted serum samples and recombinant HBeAg protein. Std serum 1, NCCL HBeAg standard serum (twofold serially diluted from 100 to 3.125 NCU/mL); Std serum 2, in-house HBV serum quantitatively calibrated using the Paul Ehrlich Institute (PEI) standard (threefold serially diluted from 555 to 0.009 PEIU/mL). (B) Comparison the analytical sensitivities of the NTR-HBeAg (16D9/14A7), Elecsys-HBeAg and Architect-HBeAg assays in detecting serially diluted WHO HBeAg reference (code: 129097/12) serum samples (threefold serially diluted from 100 to 0.046 IU/mL). (C) Comparisons of the HBeAg kinetics determined by the NTR-HBeAg (left panel) and Elecsys-HBeAg (right panel) assays in culture supernatants of HepG2 cells transfected with pHBV1.3 plasmids of various genotypes. Cell culture mediums were collected from day 0 (D0) to 10 (D10) since plasmid transfection for analysis. (D) Correlation between the NTR-HBeAg and Elecsys-HBeAg assays for all supernatant samples with various HBV genotype. GT, genotype; COI, cut-off index.

After the initial screening of mAb pairs, we found that although the 16D9 and 9F10 were both NTR-targeting mAbs, the former showed better performance with lower detecting background noise when used as capture mAbs. Therefore, we developed a novel magnetic microparticle HBeAg immunoassay using 16D9 as capture mAb. To make sure the efficient capture of HBeAg, a TG-containing sample loading buffer was introduced into the 16D9-based assays. For conjugation-mAb candidates, we found both 2A7 and 14A7 presented comparable dynamic range (about 1–1000 PEIU/mL) and analytical sensitivity in pairing with 16D9-beads (Figure 3A). The 16D9/2A7-based assay had higher analytical sensitivity for recombinant HBeAg protein but showed similar LLODs in the detection of serially diluted serum samples with the 16D9/14A7-based assay. However, in tests of a total of 464 plasma samples from HBV-free healthy donors, no reactive sample was found in the 16D9/14A7-based assay, and the specificity was 100%. In contrast, there were some false-positive samples noted in the 16D9/2A7-based assay, suggesting this mAb-pair is less specific than the 16A9/2A7 pair. Finally, we designated the 16D9/14A7-based assay as NTR-HBeAg assay and used it for further studies. In detection of serially diluted WHO HBeAg reference serum samples, the NTR-HBeAg showed a lower limits of detection (LLOD) of about 0.4 IU/mL, which was equally sensitive to the widely used Elecsys and Architect HBeAg assays (Figure 3B).

To investigate the detectability of the NTR-HBeAg assay for various HBV genotypes, we performed assay evaluation in detection of 19 commercial available human plasmas from different individuals infected with various HBV genotypes (*n* = 19, genotype A to H). In comparison to the Elecsys and Architect assays, the NTR-HBeAg assay generated similar COI values and consistent qualitative results (supplemental Table 2). The coefficients of determination (*R*^2^) between the COI values of the NTR-HBeAg assay and other two commercial assays were both over 0.99 (*R*^2^ = 0.998, *p* < 0.001 with Elecsys; *R*^2^ = 0.991, *p* < 0.001 with Architect). Next, we transfected HepG2 cells with pHBV1.3 plasmids of genotype Ae, Ba (including 2 strains of Ba (1) and Ba (2)), Ce, Cs, D, E, F, H and I. None of these HBV clones harbour G1896A mutation. The cell culture supernatants from day 0 to 10 post-transfection were collected for analysis. We compared the performance of the NTR-HBeAg and the Elecsys assays (Figure 3C). Although the HBeAg dynamic profiles of these HBV isolates were distinct, all samples collected since day 2 post-transfection were positive in both assays, suggesting the NTR-HBeAg assay was applicable for various HBV genotypes. Moreover, correlation analysis revealed that the COI values from the NTR-HBeAg assay well correlated with that of Elecsys assay (*R*^2^ = 0.913, *p* < 0.001, Figure 3D), and the two assays had similar LLOD in detecting these samples (as reflected by the regression curve going through the zero point). These results demonstrated the analytical sensitivity and the applicability of the new assay for samples with different viral genotypes.

### HBV strains harbouring G1896A could not produce detectable HBeAg in cell cultures

**Figure 4.**
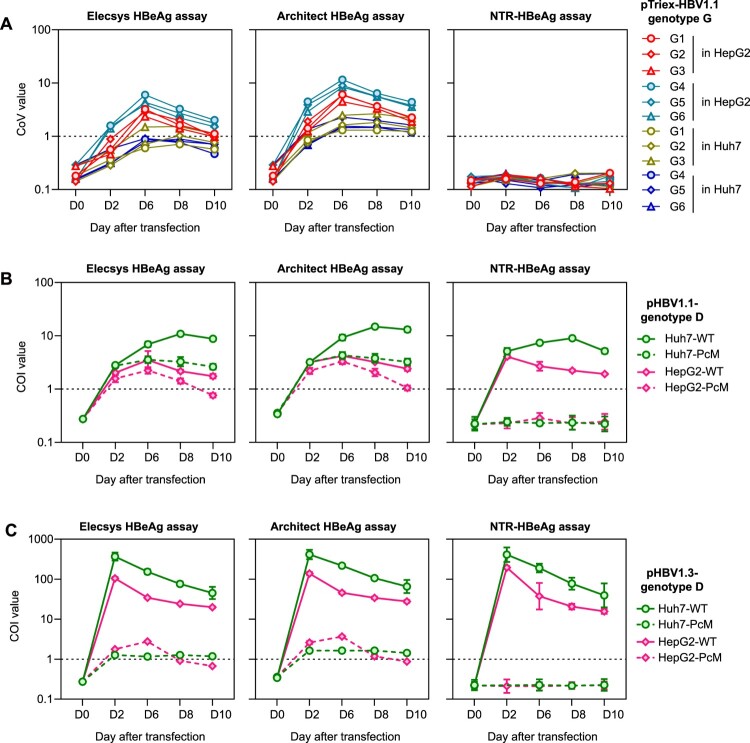
**Secreted HBcAg in cell cultures had cross-reactivity to commercial HBeAg assays but not to the NTR-HBeAg assays.** Comparisons of the HBeAg kinetics in in culture supernatants of cells (HepG2 and Huh7) transfected with pTriEx-G1 to G6 (HBV genotype G HBV strains harbouring 1817T and 1896A HBeAg-nonsense mutations) plasmids (A), or transfected with pHBV1.1 (B) or pHBV1.3 (C) plasmids of HBV genotype D with 1896G (precore wild-type, WT) or 1896A (HBeAg-nonsense mutation, PcM), using the Elecsys-HBeAg (left panel), Architect-HBeAg (middle panel) and NTR-HBeAg (right panel) assays. Cell culture mediums were collected at day 0 (D0), day 2 (D2), day 6 (D6), day 8 (D8) and day 10 (D10) for analyses. COI, cut-off index.

As HBV genotype G permanently harbours C1817T and G1896A nonsense mutations in the precore region, this genotype should be incapable of HBeAg production [24–27]. We next investigated whether the genotype G replicons in cell cultures could express detectable HBeAg by the NTR-HBeAg assay. In the experiment, six clones of genotype G (G1–G6) in pTriEx HBV1.1-form vector [24] were transfected into HepG2 and Huh7 cells, respectively. According to the study constructed these plasmids, the G1–G3 clones and G4–G6 clones were isolated from two different patients. Cell culture supernatants were collected for HBeAg detection using various assays. Compared with supernatants collected before transfection, all post-transfected samples presented elevated detection signals (COI values) in commercial Elecsys and Architect HBeAg assays (Figure 4A). Several specimens collected at day 6 (the peak) after transfection showed positive results (COI ≥ 1.0) in the two commercial HBeAg assays.

In contrast, neither detection signal elevation nor positive result was noted for these samples when detected by the NTR-HBeAg assay. Since there was no amino acid variation in the 14A7-targeting epitope for these sequences, these results suggested the HBV genotype G strains do not express HBeAg in cells. The reactive signals of these samples in the two commercial assays should be attributed to the cross-reactivity of the assay-used anti-HBe mAbs to the naked HBcAg capsid released from cells. To further validate the specificity of the 16D9/14A7 assay, we introduced precore 1896A mutation into pHBV1.3 and pHBV1.1 plasmids of a genotype D strain. The pHBV1.1 plasmid contains a 1.1-fold HBV genome under the control of CMV promoter, and viral sequences in the vectors begin at nt1817. In cells, the pHBV1.1 construct could transcribe pgRNA but not pcRNA through CMV promoter. Since the 5′ end of pgRNA does not include precore AUG codon, it would express HBcAg at a high level but could not produce HBeAg. The HBeAg in supernatants of cells transfected with pHBV1.1 would only be made from cccDNA derived from viral replication. Therefore, the pHBV1.1 construct expressed HBeAg at much lower levels than that of pHBV1.3 constructs. When tested the samples of supernatants from cells transfected with these plasmids, all three HBeAg assays presented similar positive results in those with transfection of wild-type viral sequences, in either pHBV1.1 or pHBV1.3 vectors (Figure 4B and C). However, both Elecsys and Architect HBeAg assays showed positive reactivity in samples from viral vectors with G1896A mutation, whereas the NTR-HBeAg assay exhibited completed negative results for these samples (Figure 4B and C). These results suggested that secreted HBcAg capsid has cross-reactivity on commercial HBeAg assay, but not in NTR-HBeAg assay.

### NTR-HBeAg as a surrogate marker for cccDNA in cell cultures

**Figure 5.**
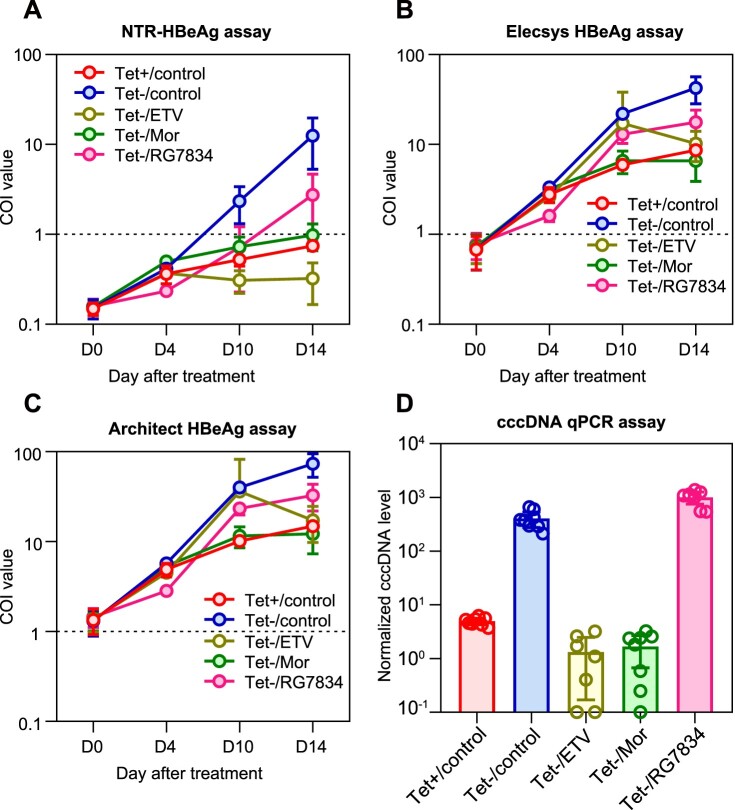
**Kinetics of HBeAg production determined by various assays and the cccDNA association in drug-treated HepAD38 cells.** HepAD38 cells were cultured and treated under the indicated conditions, culture medium were changed every 2 days and the supernatants at day 0 (D0), day 4 (D4), day 10 (D10) and day 14 (D14) since treatment administration were subjected to HBeAg measurements by the NTR-HBeAg assay (A), the Elecsys-HBeAg assay (B) and the Architect-HBeAg assay (C). At day 14 after treatment, the intracellular cccDNA levels were determined by qPCR (normalized by mtDNA quantification) and were shown in panel (D). Tet+, Tet presence; Tet-, Tet absence; ETV, Entecavir; Mor, Morphothiadin.

As previous studies had demonstrated that the HBeAg level could serve as a cccDNA surrogate in HepAD38 cell cultures [18], we next explored the practical applicability of the NTR-HBeAg assay in cell-based drug evaluations. In HepAD38 cells, viral pgRNA, cccDNA and HBeAg productions could be induced upon tetracycline removal (Tet-). We treated HepAD38/Tet- cells with three different types of HBV inhibitors, including Entecavir (ETV), Morphothiadin (Mor) and RG7834. The three drugs act as HBV inhibitors via different mechanisms: ETV is a clinical used nucleoside analogue with potent suppression effects on viral DNA replication; Mor is an HBcAg capsid assembly modulator which can block pgRNA encapsidation and subsequent synthesis of viral DNA [28]; and the RG7834 is an inhibitor for viral protein production, in particular, HBsAg expression [29]. We compared the HBeAg levels, determined by various assays, in the supernatants from HepAD38 cells treated by these drugs (Figure 5A–C). The NTR-HBeAg detection signals increased in 4 days after Tet- and exceeded the cutoff value after 10 days post Tet- (Figure 5A). At day 14 after Tet withdrawal, the average HBeAg level determined by NTR-HBeAg assay was about 12.5 COI in the untreated group (Tet-/control), whereas it was about 0.74 COI for cells with the presence of Tet (Tet+/control).

In comparison to Tet-/control group, ETV treatment (Tet-/ETV) decreased the average NTR-HBeAg level to about 0.32 COI (reduced 97%), Mor treatment (Tet-/Mor) inhibited the signal to about 0.98 COI (reduced 92%), and RG7834 treatment only showed slight reduction on NTR-HBeAg (to average 2.75 COI, reduced 78%). When examining intracellular cccDNA levels of these cells at day 14 after Tet removal and drug treatments, as expected, the ETV and Mor treatments nearly completely prevented cccDNA production in HepAD38 cells, of which both showed over 99% cccDNA reduction in comparing to Tet-/control cells (Figure 5D). Surprisingly, RG7834 treatment showed a slight cccDNA increase to control. This compound was proposed as an HBV gene expression inhibitor that mainly reduces viral antigen production, but did not show a direct suppression effect on either viral replication or cccDNA synthesis. Therefore, we speculated its promoting effects on the intracellular cccDNA level should possibly be attributed to its inhibition on HBsAg expression, as the HBsAg was identified as a negative regulator on the formation of both DP-rcDNA and cccDNA in a previous study [30]. Although the effect of RG7834 on HBeAg secretion was inconsistent with its impact on cccDNA in HepAD38 cells, the NTR-HBeAg levels were well correlated with the intracellular cccDNA levels in cells treated by other drugs. In contrast, when using conventional HBeAg assays (Elecsys and Architect assays), positive HBeAg signal appeared earlier than the 16D9/14A7 assay, but there was no significant difference between ETV group (Tet-/ETV) and Tet-/control group on either day 4 or day 10 after treatment (Figure 5B and C). At day 14 after treatment, average HBeAg reduction induced by ETV treatment was only about 75.9% and 76.6% on Elecsys and Architect assays, respectively, which were significantly lower than that presented on the 16D9/14A7 assays. Together, these results demonstrated that quantitative measurements of HBeAg in cell culture medium by the 16D9/14A7 assay could serve as a specific surrogate marker for cccDNA in HepAD38 cells.

### Performance of the NTR-HBeAg assay in detecting clinical samples

**Figure 6.**
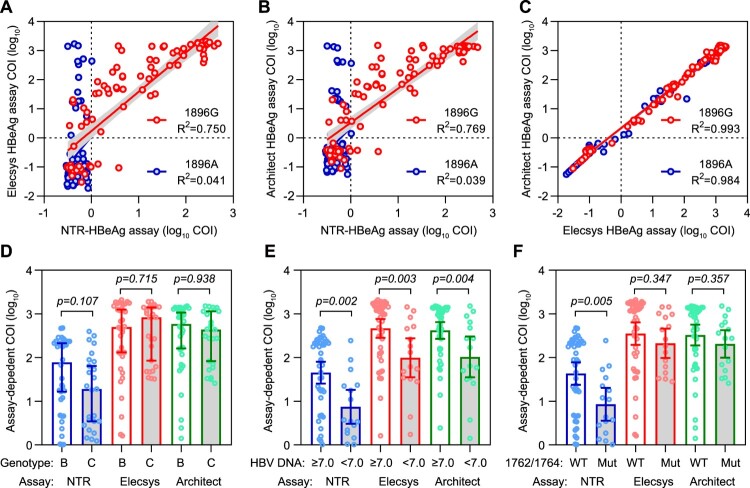
**Measurements of serum HBeAg in patients with chronic HBV infection by various assays.** The correlations between the HBeAg levels determined by three assays in patients infected with precore wild-type (1896G) or mutated (1896A) HBV viruses. (A) NTR-HBeAg versus Elecsys-HBeAg; (B) NTR-HBeAg versus Architect-HBeAg; (C) Elecsys-HBeAg versus Architect-HBeAg. Comparisons of the HBeAg levels determined by three assays in between patients divided by viral genotype (D), viral BCP mutation status (E) or viral load (F). For panel A to C, a total of 181 patients, including 91 infected with wild-type virus and 90 infected with 1896A mutated virus, were enrolled for analyses. For panel D to F, only 61 patients with precore wild-type virus and detectable NTR-HBeAg were included.

We next evaluated the performance of the NTR-HBeAg assays in detecting serum samples from a cohort consisting of 181 patients with chronic HBV infection. After viral genome sequencing, we found there were 91 patients infected with HBV harbouring 1896A mutation, whereas the remaining 90 patients infected with wild-type (1896G) HBV. Using these samples, we compared Elecsys, Architect and NTR-HBeAg assays. In samples with 1896A virus, only two showed low positivity (both with a COI value of 1.01), whereas the remaining 89 samples were negative in the 16D9/14A7 assay. In contrast, there were 17 samples with 1896A virus were positive in both Elecsys (COI value ranged from 1.23 to 1691) and Architect (COI value ranged from 1.28 to 1433) assays. Among samples with 1896G virus, correlation analyses revealed the 16D9/14A7 assay determined COI value positively correlated with data determined by Elecsys assay (*R*^2^ = 0.750, *p* < 0.001, Figure 6A) and Architect assay (*R*^2^ = 0.769, *p* < 0.001, Figure 6B). However, no association was noted between the 16D9/14A7 assay and Elecsys (*R*^2^ = 0.041, *p* = 0.053, Figure 6A) or Architect (*R*^2^ = 0.039, *p* = 0.061, Figure 6B) assay among samples with 1896A virus. In contrast, the Elecsys assay highly correlated with the Architect assay in either those with 1896G (*R*^2^ = 0.993, *p* < 0.001) or 1896A (*R*^2^ = 0.984, *p* < 0.001) virus (Figure 6C). In addition, among samples with 1896G wild-type virus, 81 (90%, including 59 positives and 22 negatives) showed consistent qualitative results in all three assays, whereas 9 samples were inconsistent (2 were NTR-HBeAg positive alone and 7 were negative in NTR-HBeAg but positive in other two assays). These results suggested the two commercial HBeAg assay may use detection antibodies with similar cross-reactivity to naked HBcAg, which may exist in some samples, in particular for those with 1896A virus.

In 61 patients with 1896G virus and detectable HBeAg in the 16D9/14A7 assay, there was no significant difference (*p* = 0.107) on HBeAg level between those infected with HBV genotype B and genotype C (Figure 6D). Similar results were also noted in two commercial assays. Whatever the detection method used, patients with a high level of HBV DNA (≥10^7^ IU/mL) had a significantly higher HBeAg level than those whose HBV DNA level <10^7^ IU/mL. In addition, among these patients, 16 were infected with HBV strain harbouring basic core promoter (BCP) mutation A1762T/G1764A [31, 32], whereas the remaining 45 patients were infected with BCP wild-type strain. Previous studies had demonstrated that the BCP mutation could reduce HBeAg production in either cell cultures or human infection. To our analysis, patients with BCP mutated viral strains showed significantly lower HBeAg levels than those infected with BCP wild-type strains when their samples were detected by the 16D9/14A7 assay (Figure 6F). In contrast, this difference was not statistically significant in the other two commercial assays.

## Discussion

In the HBV life cycle, HBeAg is not essential for viral infection, replication and cccDNA formation. However, e antigen is a pan-orthohepadnavirus conserved product which is considered as an essential immune-modulator contributing to establish persistent viral infection [33]. Clinically, HBeAg is an important diagnostic marker for CHB clinical management. Serum HBeAg status is one of the indispensable indexes for the classification of the phases of CHB natural history [3]. Serum HBeAg positivity and its high titre are associated with high-level replication of HBV and host immune tolerance to the virus, whereas HBeAg loss or seroconversion is a milestone in the natural history of chronic HBV infection and is a satisfactory short-term end-point for treatment in HBeAg-positive patients. Moreover, the HBeAg production is considered as a cccDNA surrogate in HepAD38 or HepDE19-like cell culture system, and therefore is an important tool for drug-screening targeting cccDNA [18, 30]. As the importance, immunoassays for HBeAg measurements are widely used for several years. Due to the high amino acid homology of HBeAg and HBcAg, even though the two proteins have different structures, most of anti-HBe antibodies may cross-reactive with HBcAg. The cross-reactivity of current commercial HBeAg assay to HBcAg, which is produced in non-cccDNA-dependent manner in cell culture system, greatly limits the practical applicability of this marker in the proxy measurement of cccDNA. Moreover, several previous studies found some serum samples from patients who were infected by G1896A HBeAg-minus HBV strains were positive in commercial HBeAg assays, suggesting possible false-positive detection attributed to the cross-reactivity to circulating HBcAg [34, 35].

Aiming to address the concern, in particular for cell culture system, several efforts had been made. Cai et al. developed an HepBHAe82 cell line, which carried a modified HBV construct containing an in-frame HA tag within the precore NTR region. The HepBHAe82 cells express cccDNA-dependent HA-tagged HBeAg which could be specifically detected by HA-antibody based immunoassay [36]. This system, the HepBHAe82 cells in combination with HA-HBeAg immunoassay, rules out the drawbacks of the traditional system and provides an improved drug screening system aiming cccDNA inhibition. However, some limitations should be noted. First, it requires genetic modification for involved HBV strains and is only applicable to custom cells containing engineered HBV constructs. Second, it is not applicable to clinical samples. An ideal approach to solve this problem is to develop a high-specific HBeAg assay based on high-affinity antibodies targeting the unique NTR region, which is absent on HBcAg polypeptide. Therefore, the NTR specific is essential to establish a novel HBeAg assay that completely avoids the potential interference from coexisting HBcAg. In this study, we developed two NTR-specific mAbs and one of them (16D9) exhibited high reactivity and specificity in detection HBeAg either from cell cultures or clinical samples. Our biochemical data demonstrated the 16D9 mAb recognizes the pan-genotype conserved and HBeAg-unique epitope of SKLCLG motif, which is located on aa(−10)–aa (−5) of precore protein (Figure 1D). The efficient binding of the 16D9 mAb requires the destruction of the intramolecular disulphide bridge between Cys(−7) and Cys61 [37, 38] induced by TG treatment (Figure 2C). Based on the 16D9 mAb and another FCR-mAb of 14A7, we developed a highly specific chemiluminescent microparticle HBeAg immunoassay (NTR-HBeAg). The evaluation using cell supernatants containing HBeAg produced by HBV replicon constructs of various genotype suggested the pan-genotype detectability of the new assay. More importantly, the zero cross-reactivity to HBcAg of the NTR-HBeAg assay was demonstrated by systematic testing of cell culture samples and patient's sera of G1896A viral strains (Figure 4 and Figure 6). The improved specificity of the NTR-HBeAg assay enables its applicability in cccDNA-targeting drug screening in HepAD38-like HBV cell culture system. In our proof-of-concept drug evaluation experiments, no confounding signal from cell released HBcAg capsid was observed for the NTR-HBeAg assay in detection the samples for ETV-treated cells, whereas two commercial HBeAg assay (Elecsys and Architect HBeAg assays) showed significant background (Figure 5). Overall, the NTR-HBeAg assay-derived HBeAg level showed better association with intracellular cccDNA than that derived from commercial HBeAg assays. Utilization of HBV cells with more efficient cccDNA production, like HepDES19 and Hep38.7-Tet [30, 39], in combination with the NTR-HBeAg assay may further improve the robustness of the cell-based drug screening system targeting cccDNA.

In addition to the usefulness of the NTR-HBeAg assay in detections of samples from cell cultures, we also explored its performance in serum samples from CHB patients. Nearly all samples from patients carried 1896A mutated virus had no detectable HBeAg when using NTR-HBeAg assay. In contrast, 17 of 91 samples (18.7%) from patients infected with 1896A virus were positive in two commercial assays and some of them showed high COI values (Figure 6B and C). We speculated the positivity of commercial assays in these samples may be attributed to the HBcAg cross-reactivity of their detection antibodies. This limitation may also interfere with the quantitative measurements of HBeAg in samples with precore wild-type virus (Figure 6). These results suggested the NTR-HBeAg assay may give different results in determining HBeAg status and its level of patients in comparison with the currently used commercial assay. However, it should be noted that most of the clinical samples involved in this study were from patients infected with genotype B or C, as the two genotypes were predominant in China. The significance and the influence of the NTR-HBeAg assay in clinical diagnostics and treatment response prediction requires further investigation in large-cohort studies, particularly in patients infected with various HBV genotypes. On the other hand, several factors may affect the final applications of the new assay in the clinic and research field. The current NTR-HBeAg assay was based on a fully automatic luminescence immunoassay analyser (Caris200), which can run up to 200 tests per hour and was commercially available. Although the fully automated detection procedure provides robustness and reduces safety concerns for clinical applications, it also brings additional equipment costs. Further adaptation of our mAbs to an ELISA-based assay with reduced cost may further expand the practical applicability of the method in fundamental HBV researches and drug screening studies.

In summary, we developed a novel NTR-HBeAg immunoassay using high-affinity mAbs recognizing unique NTR epitope on precore protein. The novel assay completely eliminates the confounding signal from the secreted HBcAg and therefore provides a robust tool to facilitate clinical diagnosis and drug development against HBV.
